# Challenges in obtaining accurate anthropometric measures for adults with severe obesity: A community-based study

**DOI:** 10.1177/14034948221089111

**Published:** 2022-05-01

**Authors:** Kath Williamson, David N. Blane, Michael E.J. Lean

**Affiliations:** 1 School of Medicine, Dentistry & Nursing, University of Glasgow, UK; 2 General Practice & Primary Care, Institute of Health and Wellbeing, University of Glasgow, UK

**Keywords:** anthropometry, severe obesity, bariatric care equipment, housebound people, height, weight, body mass index

## Abstract

**Aims:**

The number of people with severe obesity (BMI ⩾40 kg/m^2^) is increasing rapidly, but is poorly documented, partly as a result of inappropriate standard anthropometric measurement methods for community-based people.

**Methods:**

As part of a broader study, people receiving care services and with severe obesity were visited at home. The people were assessed for measurements using different weighing scales and a standard portable stadiometer. If the stadiometer could not be used, their half arm span and knee height were measured to estimate their height using standard predictive equations.

**Results:**

Measurements were taken for 15 women and 10 men (*n* = 25) aged 40–87 years (mean 62 years). Weights ranged from 98.4 to 211.8 kg (mean 150 kg), with 16 participants requiring bariatric scales. For the six people who were unable to stand, we used wheelchair scales (*n* = 1), bed weighing scales (*n* = 2), routine weights from care home records (*n* = 2) or weight data from hospital records (*n* = 1). The standard portable stadiometer could only be used for one person; the others required alternative measures from which to estimate height. Large body habitus obscured bony landmarks, meaning alternative measures gave diverse heights. Fourteen participants had a ⩾8 cm difference in height between estimates from half arm span and knee height measurements.

**Conclusions:**

**Standard practice commonly does not provide reliable measurements for people with severe obesity, particularly those with mobility difficulties. An inability to measure weight and height accurately can exclude people from appropriate care, obscuring the true numbers affected and the effectiveness of future service planning. Safe community care requires the availability of specialist scales and standardised methods for height estimation appropriate for older and disabled people with severe obesity.**

## Background

The public health consequences of severe obesity (body mass index (BMI) ⩾40 kg/m^2^) on premature mortality and morbidity are well recognised [1] and, more recently, the far-reaching impact of stigma on quality of care has received some attention [2]. Less well evidenced is that, despite rising numbers of adults globally with BMI ⩾40 kg/m^2^, documentation of this population is poor [3]. Some of this relates to structural issues with health surveys, such as a failure to stratify the group of people with BMI ⩾40 kg/m^2^ separately from the group of people with BMI ⩾30 kg/m^2^. However, a detailed reading of the technical reports of such surveys indicates more practical obstacles affecting data collection [4].

The problems with data collection centre on the suitability of the standard portable equipment used in the community to take anthropometric measurements for people with mobility limitations or whose weight is above the equipment’s safe working load. Currently, surveys either exclude participants unable to use standard measuring equipment or use self-report estimates [4]. Given that severe obesity is associated with functional limitations, including impaired mobility [5], these issues potentially affect the population group with BMI ⩾40 kg/m^2^ in a disproportionate manner. To date, little attention has been paid to this everyday problem and its potential impact on population studies.

At a population level, a failure to properly measure the size and nature of the growing population at the top end of the BMI scale has serious implications. People living with severe obesity – and the staff caring for them – are already struggling with care environments unable to adequately accommodate larger people [6,7], resulting in reduced quality of care or feelings of exclusion [2]. Other impacts include a lack of access to essential equipment, such as computed tomography scanners, preventing accurate diagnosis and the treatment of potentially life-threatening disease [8]. A failure to collect accurate population measurements today affects the planning and provision of services in the future. This means that care environments and equipment are failing to adapt to key population changes, continuing to exclude increasing numbers of people with severe obesity, and resulting in costly retrospective adaptations or new equipment [9].

The challenges in capturing accurate height and weight measurements from people with severe obesity are not limited to health surveys. Similar difficulties are experienced by community practitioners, such as occupational therapists and district nurses, whose role involves ordering home care equipment. An inability to record weight through a lack of suitable scales prevents access to basic care equipment. Most care equipment has a maximum weight threshold, known as a safe working load. As the number of people with severe obesity rises, these maximum weights are increasingly being surpassed, limiting access to routinely used equipment such as hospital beds, rise-recline chairs and hoists [10]. This increases both effort and the risk of harm to people with severe obesity and carers alike.

Specialist bariatric care equipment is available, albeit often at increased cost. However, access to, and use of, this equipment is dependent on a current weight to ensure safety and justify increased costs. Valid weight and height measurements are also needed by prescribers to calculate some medication doses [11] by dietitians when assessing people for weight management or nutritional interventions, and are vital to calculating BMI, in which height errors are squared [12].

Access to specialist scales is more likely in hospital settings. A hospital admission or outpatient visit provides an opportunity for an accurate weight recording, but there are time barriers to accessing the necessary equipment [10]. A lack of integration between electronic health record systems means that recordings may not be easily accessed by community or social care staff.

Height measures for people unable to stand, or older adults, are also known to be difficult to achieve accurately, with joint deformation and osteoporosis potential causes of inaccuracy [12]. This study aims to explore the challenges of taking anthropometric measures for people with severe obesity in the community, who have the potential to be excluded from standard approaches.

## Methods

The study was part of broader research into adults with severe obesity in receipt of community health and social care services. Participants were visited at their home or care home, where anthropometric measurements were taken. Participants were aged ⩾16 years and were either registered with a GP or living within the local authority area. Recruitment was via health and social care professionals involved in service provision to the relevant participants, including the lead author’s (KW) own operational contacts where applicable. This meant that most, but not all, were housebound, pragmatically defined as unable to leave their place of residence without assistance. Professionals provided brief information about the study to potential participants, gaining consent to share their contact details with KW. KW then followed up with full verbal and written information about the study. Those agreeing to participate gave written consent.

The broader mixed-methods research involved a quantitative survey, covering anthropometric measures and service utilisation, and qualitative semi-structured interviews exploring participants’ views on service provision. No weight management intervention was included. Those completing both parts were given a £10 gift voucher in recognition of their time commitment. The South East Scotland Ethics Service deemed the study to be a service evaluation. However, being part of a PhD training, the study received University of Glasgow Ethics approval. The local NHS Board Caldicott Guardian approved all data governance issues. Data collection spanned the first ten months of the SARS-CoV-2 pandemic (mid-February to end December 2020), necessitating a largely operational, pragmatic approach.

### Weight measurement

**Figure 1.**
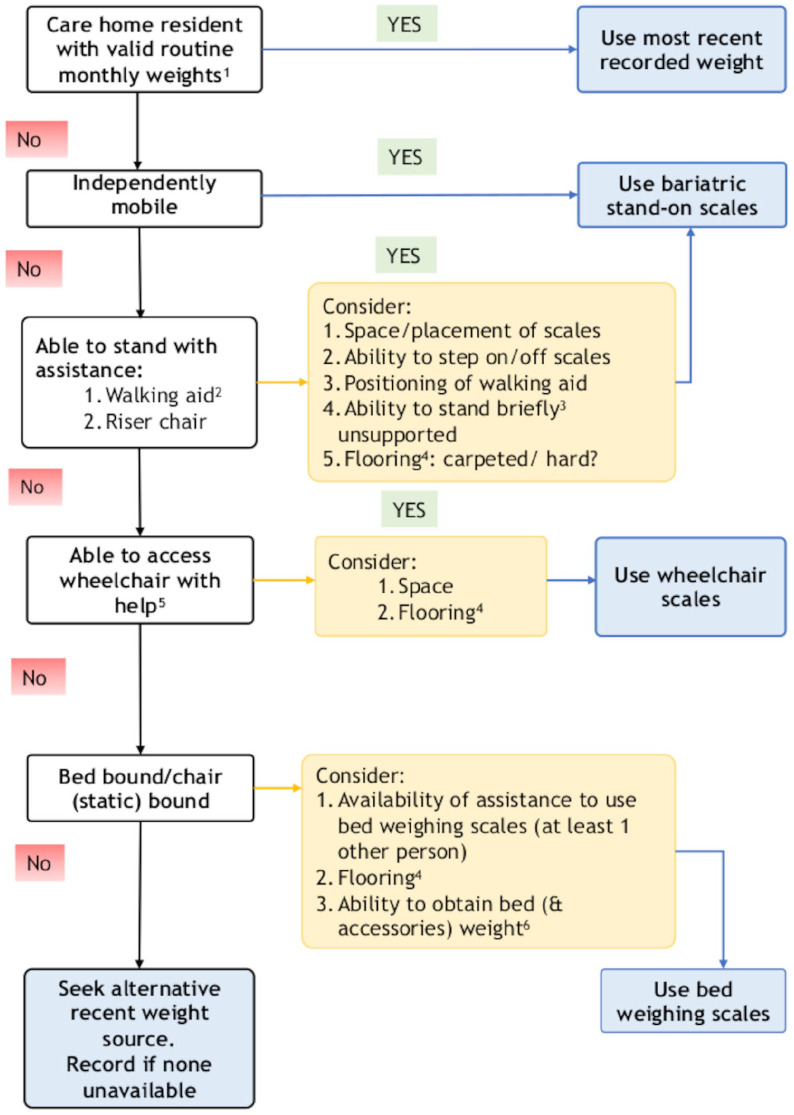
Assessment process for determining weighing scales usage. 1: Measured using calibrated chair or hoist scales; 2: stick/crutch, zimmer frame or wheeled trolley: 3: 5–10 seconds; 4: a thicker carpet reduces the accuracy of measurement while increasing manual handling risk; 5: assistance of one or two informal or formal carers; 6: scales weigh bed plus occupant; need to subtract weight of bed and accessories to obtain occupant’s weight.

**Figure 2.**
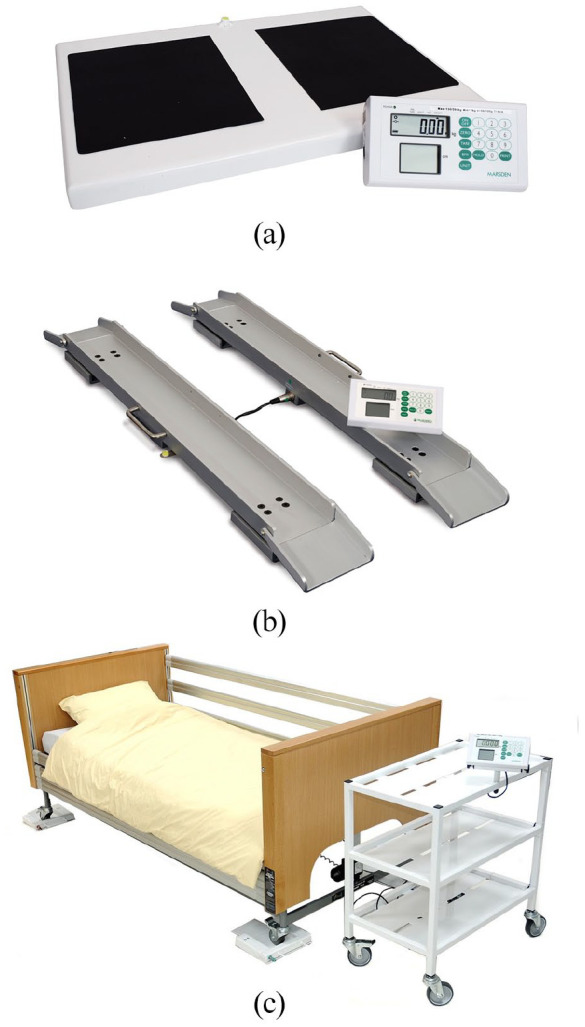
(a) M-530 high-capacity bariatric portable floor scales (width 595 mm, depth 400 mm, height 50mm; capacity 300 kg) (Marsden, Rotherham, UK). (b) Marsden M-610 portable wheelchair beam scale with two portable weighing beams, capacity 300 kg. (c) Marsden M-950 bed weighing scale (shown with hospital bed); four low-profile portable pads, capacity 1000 kg (needs 2+ operators).

**Figure 3.**
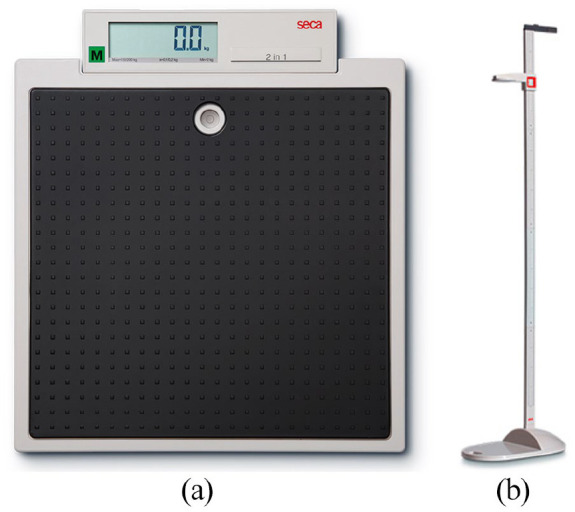
(a) Seca model 875 standard portable scales (width 321 mm, height 60 mm, depth 356 mm; capacity 200 kg) (Seca, Birmingham, UK). (b) Seca model 213 standard portable stadiometer: footplate standing area (approx) (width 337 mm, height 26 mm, depth 335 mm.

Prior to measures being taken, participants were risk assessed as per Figure 1, with outcomes informing equipment selection. If care home residents had a reliable monthly measured weight, then this was used to reduce the risk of SARS-CoV-2 transmission. The specialist scales used (Figure 2a–c) were not routinely available to community practitioners in the local area, with access negotiated for the study through weight management and manual handling services. Given that the target population of people with BMI ⩾40 kg/m^2^ has an increased risk of functional disability and falls, due both to body physique and associated comorbidities (e.g. arthritis, stroke and diabetes) [5], portable bariatric stand-on scales (M-530, Marsden, Rotherham, UK) were used for all those able to stand. These are significantly wider, deeper and lower than standard scales (Figure 3a) with a higher weight capacity. This enables access for those with wide, heavy legs, who may be unable to bring their feet close together, may be unable to see their feet when standing and may have poor balance. It also prevented stigmatising participants who may feel embarrassed or unsafe if asked to use equipment unsuitable for their size or weight [2]. If no suitable scales were identified, alternative sources of weight data were sought.

All these factors promoted safety given that the investigator was lone-working in a non-medical environment, typical of working conditions for both community practitioners and health survey interviewers.

The investigator noted participants who could have used standard scales by being (a) within the weight capacity of standard scales, (b) able to bring their feet together and (c) assessed as low risk for falls.

### Height measurement

Prior to measurement, the participants were risk assessed for their ability to stand safely on a portable stadiometer (Seca model 213, Birmingham, UK) (Figure 3b), using this if able. For those assessed as unsafe, the Medical Research Council Diet, Anthropometry and Physical Activity toolkit (DAPA) [13] outlines alternative proxy measures of half arm span (fingertip to sternal notch) and knee height. These were measured with the participant sitting or lying as able, using a steel measuring tape. Height was then estimated as double the half arm span [14] and by applying published equations for knee height [15,16].

## Results

A total of 15 women and 10 men aged 40–87 years (mean 62 years) participated in the study. Participants were largely recruited through district nursing or occupational therapy staff.

### Weight

**Figure 4.**
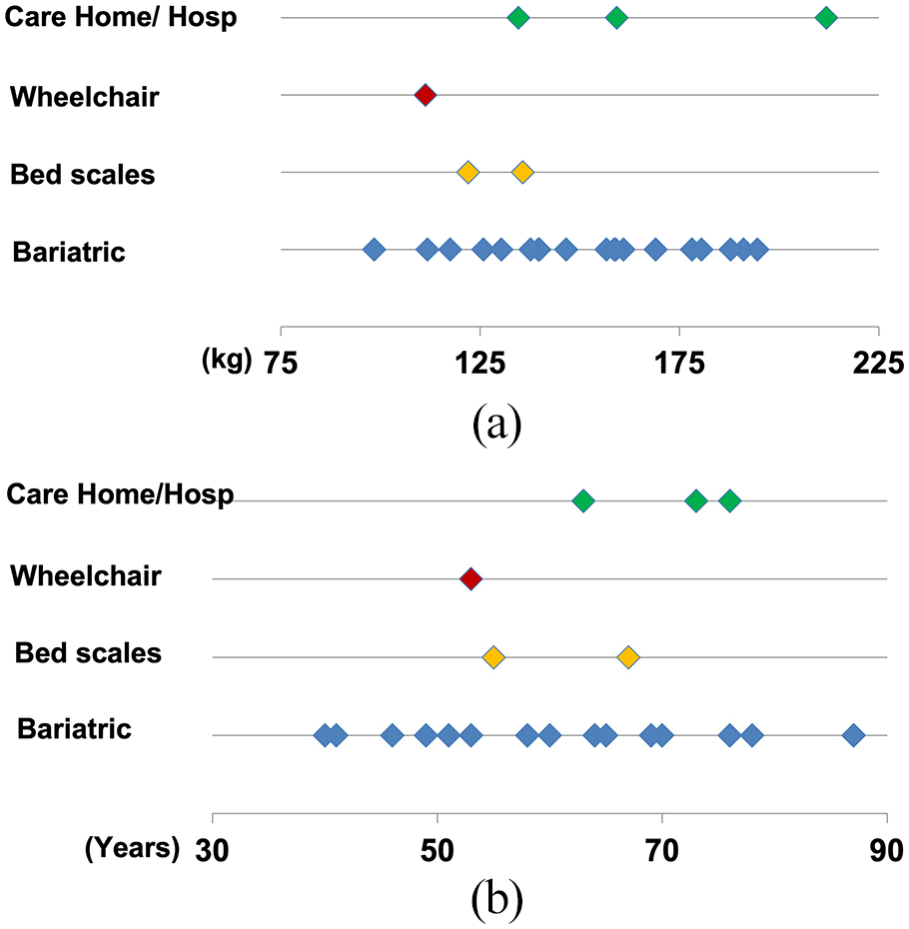
(a) Type of scales used by weight (kg). (b) Type of scales used by age (years).

Weights ranged from 98.4 to 211.8 kg (mean 150 kg) and 19 (76%) participants could stand. Of these, three (16%) were assessed as able to use standard scales and 16 (84%) required bariatric scales (largely due to either their leg size, balance or difficulty stepping on or off the narrow raised platform). Six participants were unable to stand and, of these, one used wheelchair scales and two used bed weighing scales. One bed-bound participant was unable to be weighed at home due to a lack of space and carpet in situ and therefore weight data from a hospital admission two months previously was used. Two care home residents (one bed-bound and one chair-bound) had routine monthly weights obtained using hoist or chair scales. Significantly, 80% of participants were housebound, meaning that they were unable to be weighed without specialist scales being brought to them or having a hospital admission. The type of scale used did not appear to be affected by weight (Figure 4a) or age (Figure 4b).

### Height

The portable stadiometer was difficult to use with this population and was only used for one participant. The design was a similar narrow footprint to the standard scales, with rounding at the outside edge to further decrease the platform space (Figure 3b). Participants needed to access it backwards, with the vertical height measure having a degree of movement, while not being able to provide any support. This was judged to be too risky for those with poor balance or mobility impairment. In addition, some participants had body shape characteristics, such as gluteal shelves and kyphosis, which made it difficult to stand upright against the vertical measure.

Physical limitations made alternative height measures of half arm span and knee height difficult to reliably achieve in this population, producing inconsistent results (Table I). To indicate the potential for errors between these methods of measurement and the ultimate classification for a participant, there was a mean difference of 12.3 cm, but with a standard deviation (ignoring the direction of difference) of 11.3 cm. The sample size was small, but >50% participants had a measurement error ⩾8 cm between their knee height and half arm span measures.

**Table I.** Height data comparison by participant.

Participant	Age band (years)	Sex	Half arm span (cm)	Estimated height: half arm span (cm)	Knee height (cm)	Estimated height^a^: knee height (cm)	Discrepancy between estimated half arm span and knee height (cm)	Leg bandaging in situ/gross oedema
1	40–44	F	81	162	49	159.4	2.6	N
2	40–44	F	87	174	54.5	169.8	4.2	Y
3	45–49	F	87	174	50	160.8	13.2	Y
4	45–49	F	79	158	54	168.5	–10.5	Y
5	50–54	F	81	162	41	143.7	18.3	Y
6	50–54	F	84	168	48	157.0	11	Y
7	50–54	M	88	176	55	175.3	0.7	Y
8	55–59	F	nm	nm	48	156.5	n/a	N
9	55–59	F	78	156	40	141.8	14.2	Y
10	55–59	M	104	208	63	190.3	17.7	Y
11	60–64	M	85	170	53	172.7	–2.7	Y
12	60–64	M	90	180	55	176	4	Y
13	60–64	M	85	170	49	164.3	5.7	Y
14	60–64	M	89	178	55	176.2	1.8	Y
15	65–69	F	98	196	42	145.7	50.3	Y
16	65–69	F	76	152	50	160.2	–8.2	Y
17	65–69	F	80	160	52	163.9	–3.9	Y
18	65–69	F	74	148	46	153.7	–5.7	Y
19	70–74	F	96	192	52	163.7	28.3	Y
20	70–74	M	93	186	47	159.2	26.8	Y
21	70–74	M	92	184	54.5	174.2	9.8	Y
22	75+	F	79	158	43	145.7	12.3	Y
23	75+	F	78	156	42	144.0	12	Y
24	75+	M	90	180	61	185.6	–5.6	N
25	75+	M	98	196	54	170.8	25.2	Y
Mean	62^b^		86.3	172.7	50.3	163.2	12.3	

nm: not measured (successful measurement using stadiometer, so full range alternative measures not taken).

a Knee height measure used: if <60 years mobility-impaired formula [15], if >60 years older adult formula [16].

b Age is presented as a range to promote anonymity; mean age was calculated from participant’s age in rounded years.

**Figure 5.**
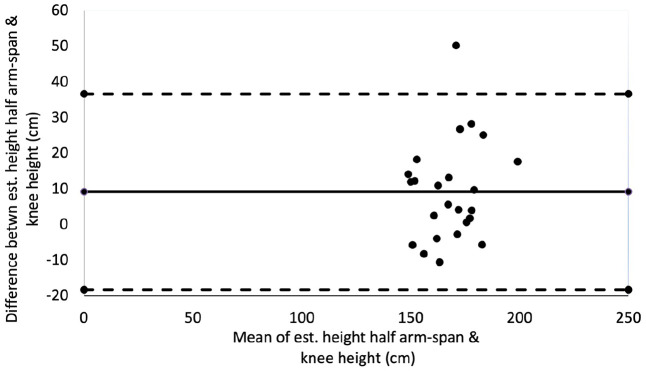
Bland–Altman plot comparing estimated height (cm) from half arm span with estimated height from knee height.

A Bland–Altman plot (Figure 5) showed no evident funnelling in the distribution of data to indicate bias between the methods. However, the wide difference between the upper and lower levels of agreement (−18.2 to +36.9 cm), equivalent to the 95% confidence intervals, demonstrates poor reliability within this population.

## Discussion

This study demonstrates the failure of standard anthropometric measuring equipment to accommodate people with severe obesity due to their larger body size, often further exacerbated by associated disability and comorbidities.

An interesting and unexpected outcome of the study was the difficulty in obtaining valid height measures, even using the alternative measures. Knee height guidance suggests the “angles of the left knee joint and ankle should be 90°” [13], with compression of the soft tissues of the heel and over the head of fibula. To the authors’ knowledge, this study is unique in recruiting via district nursing caseloads, with 22 participants having either lower limb ulceration and/or gross oedema, common with severe obesity. For these conditions, the first-line treatment is compression bandaging or hosiery toe-to-knee, shifting the build-up of fluid to around and directly above the knee joint. This directly affected both the ability to achieve a 90°angle and adequate compression of the soft tissues, precluding an accurate knee height measurement. Similarly, joint deformities, contractures and physique impeded sternal notch location and arm raising, which participants found difficult, making half arm span measures inexact.

As the study progressed, pragmatic solutions that were negotiated included taking a participant’s height against a doorpost with a tape measure (doorposts provided support for those with poor balance and were more readily accessible than wall space) (*n* = 13) [17]. If the participants were bed-bound, recumbent length measurements (*n* = 5) were attempted, but this requires someone else to assist and the participant to lie flat, which may be contraindicated due to the weight on their chest compromising breathing. Notably, several of the study participants did not use a bed, instead sleeping in a chair. If these alternative measures were exhausted, self-report was used as a last resort (*n* = 6). Current comparisons of self-reported and measured height using large samples show both men and women overestimate height [18]. Although the overall mean difference between measures may be small at <1.5 cm, it potentially leads to misclassification by lowering the BMI [18].

Our findings are consistent with research among service providers from hospitals in Ireland [10] and rural practices in the USA [19], highlighting that the lack of provision of scales for those with high weight is itself a barrier to being weighed. Health surveys for both Scotland and England currently use only standard class III portable scales. Given that functional disability increases with BMI class, this is likely to affect the population with BMI ⩾40 kg/m^2^ more than other BMI categories, resulting in under-documentation.

Much previous research exploring alternative height measures has limited application to this study group. A large body habitus, along with related comorbidities, frequently prohibits accurate measures of relevant body parts. A previous study by Hickson and Frost [14] similarly found these measures restricted by a person functionality and comorbidities in an acutely ill elderly population, a reason why height is often not performed as part of nutritional assessment [14]. They concluded that there was no ideal surrogate measure, cautioning that within-group comparison should use the same measure. Likely reasons for non-agreement are that older people tend to lose height and measured height may fall with kyphosis or hip and knee arthritis, and arm span reflects maximal adult height rather than true (current) height [14]. The terms half arm span and demi span are interchangeable from a linguistic perspective [13], but some papers have used these terms to refer to different measures, using extended fingertips or finger root, requiring the application of the correct calculations [14]. Other studies are restricted to healthy populations [12], actively exclude those with high BMI [20], or consider a disability affecting only one body part, e.g. spinal [20], leaving other viable alternative measures available.

Ulna length, included in the British Association for Parenteral and Enteral Nutrition (BAPEN) Malnutrition Universal Screening Tool (MUST) [21] to estimate height for both men and women <65 years and >65 years, was not considered for the present study because it is not included in DAPA’s alternative measures [13]. Its focus is undernutrition, so its application to those with BMI ⩾40 kg/m^2^ is unexplored. Ulna length faces the same issues of locating body landmarks (specifically the midpoint of the styloid process) due to large body habitus, but to a reduced degree, as the forearm suffers less from gross oedema or the joint problems common in lower limbs [22]. Thus, despite evidence suggesting that ulna length is not the most accurate measure in other populations [12], ulna length appears to offer the greatest potential for accuracy and ease of measurement with this population. This applies even for those with mobility difficulties in community settings, making it an obvious choice for use and further research in this population.

This study provides novel evidence on the challenges of accurate height and weight measurement from a hard-to-reach, largely undocumented population. Although evidence exists in populations with BMI ⩾40 kg/m^2^, these focus on people receiving weight management treatment [23], including bariatric surgery [24] or hospital-based service utilisation [25]. There is minimal evidence looking at non-medical or community service utilisation, especially research considering the needs of a housebound population. This reflects difficulties in accessing samples, data collection and the emerging nature of this population.

The participants available and willing to be measured for this study are not a nationally representative sample, but as exemplar cases they offer insights about a population for whom little evidence exists to guide policy-makers or care providers. They reflect an important subset of people with BMI ⩾40 kg/m^2^ most affected by functional disability and therefore with high potential for using health and care services. Standard surveys or secondary research using routine data or population health studies commonly fail to reach this population for the reasons already highlighted. Functional disability is seldom wholly attributable to high body weight because other factors, such as arthritis, become involved. However, high body weight exacerbates such disability rather than reduces it. Further research disaggregating the wider BMI ⩾40 kg/m^2^ population into BMI ⩾50/60/70 kg/m^2^ subsets, or by functional status, might enable improved care provision.

The study evidences the challenges faced by health and social care practitioners when taking anthropometric measures from people with severe obesity in the community. Indeed, there was anecdotal evidence that local practitioners referred people into the study as a means of obtaining their weight due to a lack of local provision. People outside clinical services might assume practitioners have ready access to specialist scales. Paradoxically, it is the very lack of measurement that leads to this population’s lack of visibility. What is not measured cannot be evidenced, making it difficult to quantify the need for these scales to managers with constrained resources. This resulting under-documentation is concerning given the use of health survey data to inform future service planning and provision [3]. Failure to fully document the population shift of increasing numbers in the right tail of the BMI distribution curve hinders effective modelling of future population projections [26].

Substantial planning and adaptation are needed to accommodate larger people with associated equipment needs, such as tracking hoists, larger rooms to accommodate equipment [27] and more staff to facilitate care [28]. The implications of these findings are not limited to care services, applying to the design of multiple diverse contexts and environments, including transport, crematoriums, housing, and fire and rescue services. Consequently, there is high potential for failure to accommodate affected people without significant and costly retrospective changes to buildings, vehicles, equipment and staff training. Serious implications for people [2,8] and staff [6,19] are already evident and likely to worsen.

The need for specialist scales appears to be a gap in the evidence base, particularly affecting, but not limited to, community settings, including health surveys. The procurement of bariatric stand-on scales by non-specialist services is recommended as an initial first step to promoting inclusion, while being relatively cheap and simple to use. Providing access to wheelchair or bed weighing scales is more complex due to the increased costs and need for training and risk assessment, but needs to be developed.

## Conclusions

Standard anthropometric methods commonly do not provide reliable measurements for people with severe obesity, particularly those with mobility difficulties. Failure to measure weight and height accurately can exclude people from appropriate care, obscures the true numbers affected, and thus impacts service provision and planning. Safe community care requires the availability of specialist scales and training in the use of standardised methods for height estimation appropriate for use in older and disabled people with severe obesity.
